# Iron, Cobalt, and Nickel Phthalocyanine Tri-Doped Electrospun Carbon Nanofibre-Based Catalyst for Rechargeable Zinc–Air Battery Air Electrode

**DOI:** 10.3390/ma16134626

**Published:** 2023-06-27

**Authors:** Kaur Muuli, Rohit Kumar, Marek Mooste, Viktoria Gudkova, Alexey Treshchalov, Helle-Mai Piirsoo, Arvo Kikas, Jaan Aruväli, Vambola Kisand, Aile Tamm, Andres Krumme, Prabu Moni, Michaela Wilhelm, Kaido Tammeveski

**Affiliations:** 1 Institute of Chemistry, University of Tartu, Ravila 14a, 50411 Tartu, Estonia; 2 Department of Materials and Environmental Technology, Tallinn University of Technology, Ehitajate tee 5, 19086 Tallinn, Estonia; 3 Institute of Physics, University of Tartu, W. Ostwald Str. 1, 50411 Tartu, Estonia; 4 Institute of Ecology and Earth Sciences, University of Tartu, Vanemuise 46, 51014 Tartu, Estonia; 5 Advanced Ceramics, University of Bremen, Am Biologischen Garten 2, IW3, 28359 Bremen, Germany

**Keywords:** electrospinning, carbon nanofibres, non-precious metal catalyst, carbon nanomaterial, metal phthalocyanine, bifunctional catalyst, electrocatalysis, zinc–air battery, oxygen reduction

## Abstract

The goal of achieving the large-scale production of zero-emission vehicles by 2035 will create high expectations for electric vehicle (EV) development and availability. Currently, a major problem is the lack of suitable batteries and battery materials in large quantities. The rechargeable zinc–air battery (RZAB) is a promising energy-storage technology for EVs due to the environmental friendliness and low production cost. Herein, iron, cobalt, and nickel phthalocyanine tri-doped electrospun carbon nanofibre-based (FeCoNi-CNF) catalyst material is presented as an affordable and promising alternative to Pt-group metal (PGM)-based catalyst. The FeCoNi-CNF-coated glassy carbon electrode showed an oxygen reduction reaction/oxygen evolution reaction reversibility of 0.89 V in 0.1 M KOH solution. In RZAB, the maximum discharge power density (*P*_max_) of 120 mW cm^−2^ was obtained with FeCoNi-CNF, which is 86% of the *P*_max_ measured with the PGM-based catalyst. Furthermore, during the RZAB charge–discharge cycling, the FeCoNi-CNF air electrode was found to be superior to the commercial PGM electrocatalyst in terms of operational durability and at least two times higher total life-time.

## 1. Introduction

Recently, governments of different countries around the world have set a goal for all new cars and vans registered to meet the criterion of zero-emission by 2035 [1]. This agreement will favour the ever-growing worldwide demand for sustainable and affordable electric vehicles (EVs). A drawback for the large-scale worldwide introduction of EVs is the lack of cheap and environmentally compatible batteries and battery materials. Due to their environmental friendliness, high energy density, and low production cost, rechargeable zinc–air batteries (RZABs) are one of the most promising next-generation energy-storage technologies for EVs and also for stationary applications [2,3,4]. RZABs have been also proposed as a compelling alternative to Li-ion battery technology based on rechargeability, safety, and cost issues [5,6]. However, despite the theoretically high performance and economic potential, the development of practically viable RZABs is still a challenge due to issues like zinc irreversibility, optimal battery configuration, and efficiency to catalyse the two key oxygen-related reactions [3,7].

One of these key reactions is the oxygen reduction reaction (ORR) occurring at the RZAB air electrode during battery discharge. The ORR in alkaline solution can proceed via a 2e^−^ or 4e^−^ pathway, although the 4e^−^ route is preferred to avoid the peroxide production and due to the higher energy efficiency per O_2_ molecule [5]:

O_2_ + 2H_2_O + 4e^−^→4OH^−^ (1)

While charging the battery, the reverse reaction, i.e., the oxygen evolution reaction (OER), takes place at the RZAB air electrode [8]:

4OH^−^→2H_2_O + O_2_ + 4e^−^ (2)

The high overpotential and sluggish kinetics of both reactions, ORR and OER, limit the commercialization of ZABs [9,10]. As a solution, high-performance bifunctional oxygen catalysts can significantly minimize the overpotentials of the ORR and OER, e.g., Pt-group metal (PGM)-based catalysts [7]. As PGM-containing materials are limited resources and expensive, it is of great importance to develop efficient and low-cost non-PGM bifunctional catalysts [7,9,11]. In recent years, carbon nanomaterials with high specific surface area and electrical conductivity (e.g., carbon nanotube [CNT], carbon nanofibre [CNF], carbon nanosheets, and graphene family materials) have been increasingly investigated as alkaline RZAB air electrode catalyst support materials [8,12].

Electrospun CNFs with unique properties such as high porosity, flexibility, strength, and high specific surface area possess great advantages by having the space for a large number of active sites, shortening the diffusion pathways, and improving the reaction kinetics, which makes them promising for RZAB air electrode catalyst application [5,9]. Furthermore, this technique enables versatile introduction of different transition metals (TMs) and their compounds into CNFs, which have been confirmed to provide the active sites for ORR (e.g., TM-Nx) and OER (e.g., TM oxides) [8,9,13,14]. Despite the problems with uniform distribution of several TMs at once in one nanocarbon catalyst, multiple TM-containing materials have been proposed to be more efficient non-PGM bifunctional oxygen catalysts than single TM-based ones [15,16,17,18,19,20,21,22]. In addition to dual TM-embedded CNF catalysts employed for RZAB air electrode application [23,24,25], triple TM combination with various nanocarbon supports have been also investigated [26,27,28,29,30,31,32,33]. A very appealing and effective approach to introduce TM-Nx active sites into nanocarbon catalysts is the inclusion of TM macrocyclic compounds (e.g., metal phthalocyanines (Pc) or porphyrins) [34,35,36,37,38,39,40,41,42]. Different TM Pc compounds have been also found to be efficient ORR and OER bifunctional catalysts according to computational studies [43]. Furthermore, in a recent investigation, a CNT-based catalyst dual-doped with FePc and NiPc was successfully implemented as an air electrode material in RZAB application, showing better long-term cycling stability and higher maximum discharge power density compared to the commercial Pt-Ru/C catalyst [37]. To our knowledge, there is no information available for the application of electrospun CNF materials embedded in dual- or triple-doped combinations with NiPc, FePc, and CoPc compounds as a bifunctional oxygen catalyst in the RZAB air electrode.

The aim of this investigation is the first-time preparation of triple metal Pc (NiPc, FePc, and CoPc) embedded CNF bifunctional catalyst via pyrolysis for ZAB application. The influence of carbonisation temperature and acid treatment conditions on the ORR and OER activity of the prepared materials is studied in 0.1 M KOH. The catalyst material with the highest ORR and OER activity is employed as an air electrode catalyst in primary and secondary ZAB accompanied by the comparison with commercial Pt-Ru/C catalyst.

## 2. Materials and Methods

### 2.1. Material Preparation

Three electrospinning solutions with different composition and concentration of the ionic liquid (IL) and TM phthalocyanines were prepared for electrospinning of fibrous membranes, which were later used for RZAB air electrode catalyst preparation. Firstly, 10 wt% of polyacrylonitrile (PAN, MW = 150,000, Sigma Aldrich, St. Louis, MO, USA) polymer solution in N,N-dimethylformamide (DMF, Sigma Aldrich) was prepared by mechanical mixing at 40 °C for 24 h. After that, the IL of 1-butyl-3-methylimidazolium acetate ([Bmim]Ac, Sigma-Aldrich), cobalt(II) phthalocyanine (CoPc, Alfa Aesar, Haverhill, MA, USA), iron(II) phthalocyanine (FePc, Acros Organics, Geel, Belgium), and nickel(II) phthalocyanine (NiPc, 95%, Alfa Aesar) were added in a glovebox under N_2_ atmosphere (O_2_ content was held below 1%) and mechanically mixed at room temperature for another 24 h. The concentration of FePc, CoPc, and NiPc in the final electrospun membrane (Table 1) was chosen according to the TM Pc to PAN ratio that produced the FePc, CoPc dual-doped and IL-containing material-based catalyst with the highest ORR activity in our previous investigation [44]. The use of higher amounts of TM Pc in the solution would introduce problems with efficient electrospinning.

Prepared solutions were electrospun at room temperature and ambient air conditions in an electrospinning cabinet from a disposable 5 mL plastic syringe connected to a stainless-steel needle (inner diameter of 0.6 mm). Power supply (MK Series) was used to generate a high voltage up to 17 kV. For FePc- and NiPc-containing solution, the applied voltage (AV) was between 15 and 17 kV. For the other two solutions, the AV was from 12 to 17 kV. Fibres were collected in a rotating drum collector, covered with aluminium foil. The diameter of the drum was 7.5 cm, and the rotating speed was constantly kept at 4500 rpm. The distance to the collector and the solution feed rate for the FePc- and NiPc-containing solution were 6–8 cm and 0.9–1.5 mL/h, respectively. For the two other solutions, the feed rate and distance to collector were 0.4–0.6 mL/h and 10–12 cm, respectively. An NE-1000 Single Syringe Pump (New Era Pump Systems Inc., Farmingdale, NY, USA) was used to pump the electrospinning solutions. After the electrospinning, the fibrous membranes were subjected to pyrolysis at 800 °C in N_2_ atmosphere according to the procedure described in our previous paper [44]. The CNF catalysts were obtained after carbonisation, and have TMs in the designation referring to the specific TM Pc used in the electrospinning solution (Table 1). If a pyrolysis temperature other than 800 °C was used, this is additionally marked in the catalyst designation. Also, if acid leaching was employed for the catalysts, this is marked in the material name as 1M or 3M according to the molarity used for the aqueous H_2_SO_4_ and HNO_3_ acid mixture. The specific procedure for acid leaching is adapted from our previous investigation [44].

### 2.2. Physical Characterization

Morphology of catalysts was characterized with scanning electron microscopy (SEM), and elemental composition was determined with energy dispersive X-ray spectroscopy (EDX). The Si plate coated with the catalyst ink prepared in 2-propanol (99.8 %, Honeywell Riedel-de Haën, Berlin, Germany) was employed for the SEM-EDX characterization. For SEM-EDX measurements, an FEI Helios NanoLab 600 microscope (Hillsboro, OR, USA) with an Oxford Instruments INCA Energy 350 EDX detector was used (Abingdon, UK). Elemental distribution was studied with an FEI Titan Themis 200 scanning transmission electron microscope (STEM) equipped with Bruker SuperX EDX detectors (Billerica, MA, USA).

X-ray photoelectron spectroscopy (XPS) studies were performed with an SCIENTA SES-100 spectrometer using 200 eV pass energy (Taunusstein, Germany). A glassy carbon (GC) plate coated with the catalyst ink prepared in 2-propanol was employed for the XPS experiments. The electron take-off angle was 90°. The non-monochromatic Mg K_α_ X-ray photons (with energy 1253.6 eV) were used for excitation. The pressure inside the analysis chamber was below 10^–9^ mbar during data collection. The raw data were processed using Casa XPS software (version 2.3.17). Data processing involved removal of X-ray satellites, and peak fitting using the Gauss–Lorentz hybrid lineshapes and combination of linear and Shirley backgrounds.

X-ray diffraction (XRD) analysis was carried out with the catalysts in powder form using a Bruker D8 Advance diffractometer with Ni-filtered Cu K_α_ radiation and a LynxEye line detector. The diffraction patterns were collected using a scanning step of 0.013° 2θ and a counting time of 534 s per step in a range of 10 to 92° 2θ. The ICDD PDF-4+ (2021) database and the Crystallography Open Database were used to identify the crystalline phases.

The micro-Raman spectroscopic technique was used to characterize the catalyst materials. All samples were dispersed in 2-propanol by sonication and drop-coated onto Si substrates. Raman spectra were recorded in the back-scattering geometry on an inVia Renishaw spectrometer (Wotton-under-Edge, UK) in conjunction with a confocal microscope (Leica Microsystems CMS GmbH, Wetzlar, Germany), 50× objective, and an argon ion laser operated at 514.5 nm. Laser-induced overheating and photochemistry effects were avoided by decreasing the laser power to 2 mW and defocusing the laser spot on the sample to about 20 μm. The spectra processing involved a baseline correction and multiple-peak fitting procedure using the PeakAnalyser software in OriginPro 9.5.1.

### 2.3. Electrochemical Characterization

The electrochemical studies were performed in Ar-saturated (99.999%, AS Linde Gas, Tallinn, Estonia) or O_2_-saturated (99.999%, AS Linde Gas) 0.1 M KOH (90%, Lach-Ner, Neratovice, Czech Republic). The catalyst ink consisted of 8 mg of non-precious metal catalyst (NPMC), 2 mL of 2-propanol, and 10 μL of Nafion^®^ dispersion (5 wt%, Aldrich). The ink was sonicated for ~90 min and drop-cast onto the polished and cleaned [45] GC (*A* = 0.196 cm^2^) electrodes (GC-20SS, Tokai Carbon, Tokyo, Japan), with final catalyst loading of 0.2 mg cm^−2^. A three-electrode system was used with the NPMC-coated GC as the working electrode, carbon rod as a counter electrode, and saturated calomel electrode (SCE) as a reference electrode. The reported potentials were converted from vs. SCE to vs. reversible hydrogen electrode (RHE) [46]. Cyclic voltammetry (CV) and rotating disc electrode (RDE) methods were employed for the electrochemical measurements using an Autolab potentiostat/galvanostat PGSTAT30 (Metrohm Autolab, Utrecht, The Netherlands) and Nova 2.1 software. The RDE measurements were performed using an EDI101 rotator and a CTV101 speed control unit. The polarisation curves for ORR were recorded in the cathodic direction, while the polarisation curves for OER were recorded in the anodic direction. The ORR and OER polarisation curves were IR compensated using the data obtained with electrochemical impedance spectroscopy [46].

### 2.4. Zn–Air Battery Testing

The in-house built device [19,47] was employed to study the catalyst’s performance and stability in (rechargeable) ZAB conditions. The electrolyte in the primary and rechargeable ZAB was 6 M KOH solution containing 0.2 M Zn(CH_3_COO)_2_. For the catalyst ink preparation, 7 mg of FeCoNi-CNF was dispersed in 800 µL of water:ethanol mixture (volume ratio 1:3) containing 20 μL 5% Nafion solution via sonication for 60 min. For the preparation of the air electrode, the catalyst ink was drop-cast onto a 3.5 cm^2^ gas-diffusion layer (Sigracet 39 BB, SGL Carbon, Wiesbaden, Germany) to obtain FeCoNi-CNF loading of 2 mg cm^−2^. For comparison, the Pt-Ru/C (50:25:25, Alfa Aesar) catalyst with 1 mg cm^−2^ loading was also employed. The geometric area of the air electrode exposed to the electrolyte solution was 0.79 cm^2^. As the counter electrode, polished zinc foil (99.9%, 0.2 mm thickness, Auto-plaza) was used with a geometric area of 2.55 cm^2^ exposed to the electrolyte solution. The electrode areas were limited and made leak-proof using acrylonitrile butadiene rubber o-rings. The polarisation curves for primary battery were recorded in galvanostatic mode using a scan rate of 10 mA min^−1^ until a cell voltage less than 0.2 V was achieved.

## 3. Results and Discussion

### 3.1. Physical Characterization

**Figure 1:**
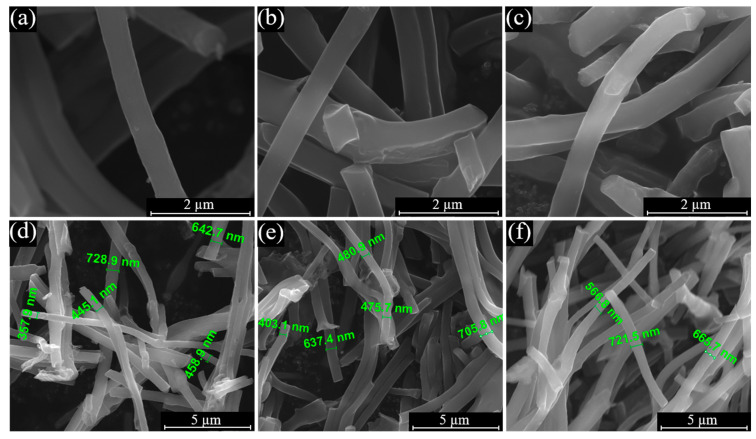
SEM micrographs of (**a**,**d**) FeNi-CNF, (**b**,**e**) CoNi-CNF, and (**c**,**f**) FeCoNi-CNF samples.

In Figure 1, SEM images for electrospun fibre-based catalysts with different composition prepared via pyrolysis at 800 °C are shown. All three CNF materials are similar, possessing a smooth surface, diameters in the range of 350–750 nm, and variations in length. Adhesion between aligned fibres occurred; however, general orientation seemed random. The fibre diameter is similar to the ones of Fe/Co/IL-CNF-800-based catalysts, which were prepared in our previous investigation using Fe and Co acetate salts as TM precursors for ORR electrocatalyst application [48]. The observed nanofibrous structure is known to be beneficial for surface reactions (e.g., ORR and OER electrocatalysis), as it provides high specific surface area available for active sites with respect to the material weight and also shorter pathways for mass transport due to the nanoscale dimensions [49,50,51]. After the acid treatment procedure, which was followed by second pyrolysis, a difference in the surface roughness is observed (Figure S1 in Supplementary Materials). More specifically, the surface of FeCoNi-CNF-1M has formations similar to the ones observed for the IL and PAN-based CNF materials in our earlier study prepared using Fe and Co acetate salts as TM precursors [48].

**Figure 2:**
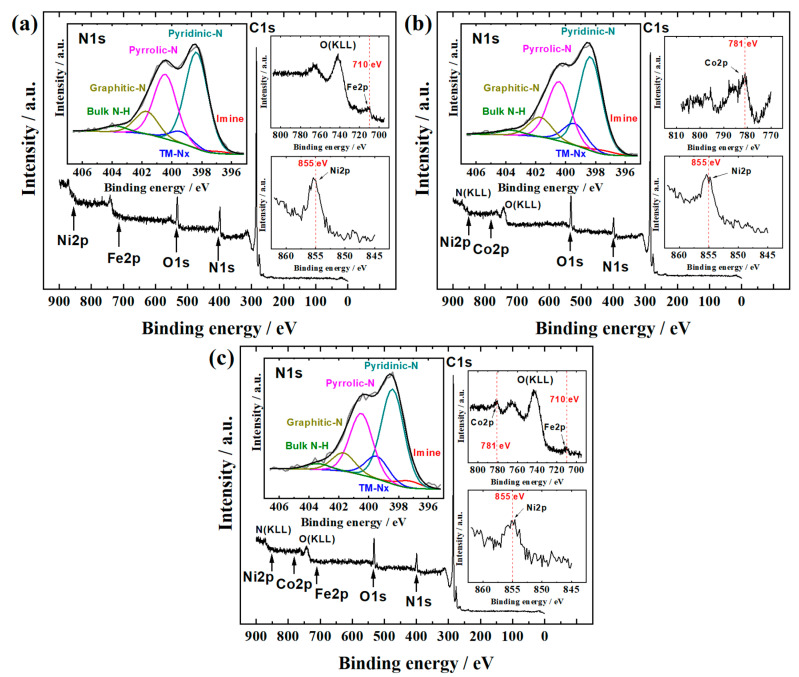
XPS survey spectra of (**a**) FeNi-CNF, (**b**) CoNi-CNF, and (**c**) FeCoNi-CNF samples. The insets show detailed spectra in the N1s region with deconvoluted peaks and in the Fe 2p, Co 2p, and Ni 2p regions.

Studying the surface elemental composition is beneficial for investigating the ORR and OER activity of CNF-based catalysts. According to the XPS measurements (Figure 2 and Table 2), the major components in the case of all three CNF materials are carbon (C 1 s at 285 eV), oxygen (O 1 s at 533 eV), and nitrogen (N 1 s at 400 eV), as is expected for the electrospun PAN-based catalysts from previous investigations [45,48]. Regarding TM Pc incorporation, the components of N, Fe, Co, and Ni should be considered the most important. The low at% content and high noise level of the XPS signal in TM 2p regions makes it difficult to exactly find out the chemical states of TMs on the catalyst surface, but the XPS peak maxima can be still observed. For comparison, the Ni 2p XPS peak centred at 855 eV is known to appear in the NiPc-functionalized nanocarbon catalysts [37,52]. Also, the Fe 2p and Co 2p XPS peaks have been previously reported to be around 710 and 781 eV, respectively, for corresponding Pc-compound embedded nanocarbon materials [37,53,54,55,56,57]. Additionally, these XPS peak maximum values reported herein for Fe 2p, Co 2p, Ni 2p regions most likely correspond to the oxidized states of corresponding TMs [48,52,58], which is in accordance with the literature as TM(II) have been claimed to be the species in TM-Nx site to provide high ORR activity [13,59,60,61]. Therefore, the registered photoelectrons of these metals most likely correspond to the TM centres in the TM-Nx sites. In the deconvoluted N 1s spectra, the TM-Nx component is also observed at ca. 399.6 eV, which is accompanied by other major components such as pyridinic-N, pyrrolic-N, graphitic-N. In general, the synergy of all these different N-species has been considered beneficial for ORR electrocatalysis in the case of nanocarbon catalysts [62,63,64]. While the graphitic-N and Co-Nx catalyse the 2e^−^ ORR from O_2_ to HO_2_^–^, the pyridinic-N, pyrrolic-N, and Fe-Nx catalyse the 4e^−^ route including the further reduction of HO_2_^–^ to the final product of OH^–^ [54,65]. For OER, the detection of Fe 2p, Ni 2p, Co 2p, and O 1s XPS peaks is very important, as TM oxides are known to be OER-active sites with high activity and stability in alkaline conditions [66,67,68]. Moreover, the Fe, Co, and Ni oxides have been found to be present in the composition of nanocarbon catalysts prepared via pyrolysis with corresponding TM Pc compounds [37,40].

**Figure 3:**
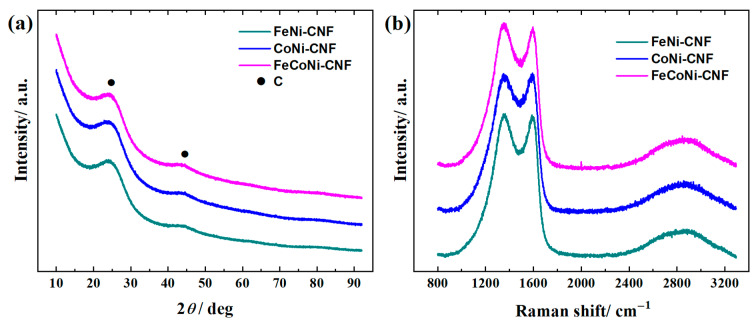
(**a**) XRD patterns and (**b**) Raman spectra for FeNi-CNF, CoNi-CNF, and FeCoNi-CNF catalyst materials.

XRD analysis can be employed to investigate the structure of the nanocarbon catalyst (Figure 3a). As expected for the CNF-based catalysts, the XRD peaks at 23 and 44°, which correspond to the (002) and (100) reflections of graphitic carbon, respectively, are noted [40,69]. According to the XRD data, there is no evidence for the formation of TM alloys [23,25], nanoparticles [66,69], or oxides [70,71] in a quantity that would be detectable. This indicates the presence of well dispersed ORR and OER active sites in the CNF catalyst with single-atomic nature (e.g., Co-Nx, Fe-Nx, Ni-Nx) rather than to the agglomeration of TM components into detectable nanoparticles [69]. Raman spectroscopy was used to gain further insight into the carbon backbone structure of the CNF catalyst. The Raman spectra of the materials normalized to the intensity of the G band are presented in Figure 3b. The Raman spectra of three catalysts are rather similar to each other and also to the previous PAN-based CNF [48] and TMPc functionalized carbon materials [71]. The first-order Raman spectra were fitted following the four-peak model, where the G peak (1590 cm^−1^) corresponds to the stretching vibrations of the sp^2^ carbon atoms in the ideal graphitic lattice; D1 (1356 cm^−1^) corresponds to defect-activated breathing mode of aromatic rings; D3 (1495 cm^−1^) to amorphous carbon; and D4 (1160 cm^−1^) to disordered graphitic lattice [72]. The *I*_D1_/*I*_G_ ratio (integrated areas under the bands) were ca. 1.8–1.9 for all three samples. The strong broadening of D1 and G bands indicates the presence of defective carbon structures due to the functionalization procedure. The region of the second-order Raman spectra consists of suppressed and highly broadened 2D1 (~2700 cm^−1^) and D1 + G (~2940 cm^−1^) bands, similar to severely defective few-layer graphene material [73].

**Figure 4:**
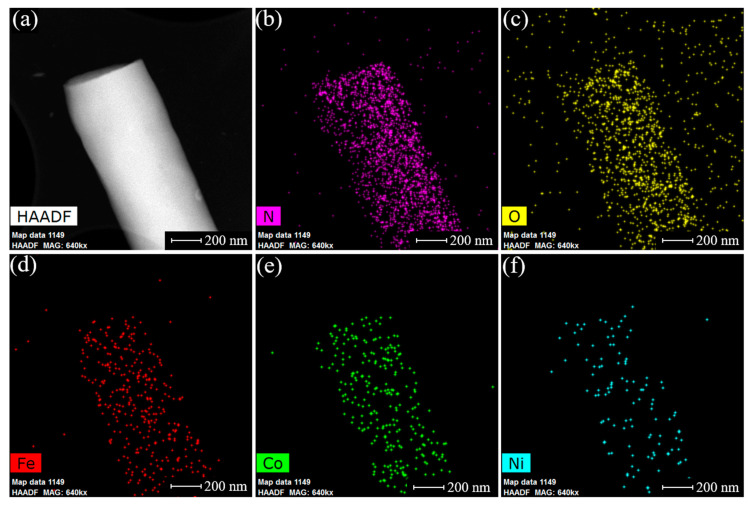
(**a**) STEM image and EDX elemental maps of (**b**) nitrogen, (**c**) oxygen, (**d**) iron, (**e**) cobalt, (**f**) nickel for FeCoNi-CNF catalyst.

As the main aim of this paper is the preparation of FePc, CoPc, and NiPc tri-doped CNF bifunctional catalyst, the elemental distribution and composition of FeCoNi-CNF was studied in more detail. The uniform distribution of elements on a nanometre scale was found according to the STEM-EDX mapping as shown in Figure 4, indicating the presence of well-dispersed TM-Nx sites of single-atomic nature rather than agglomerated nanoparticles. To investigate the bulk elemental composition of FeCoNi-CNF catalyst, SEM-EDX measurement was performed with the following material content by weight: 68% C, 17.5% N, 11.5% O, 0.7% Ni, 1.1% Fe, and 1.2% Co. According to the latter result, the bulk amount of each TM in the FeCoNi-CNF material is around 1 wt%. Furthermore, the acid treatment procedures for FeCoNi-CNF had no clearly identifiable influence on the TM content according to SEM-EDX analysis, which is consistent with the report by Buan et al. claiming that TM-Nx species are resistant to the acid leaching [74]. 

### 3.2. ORR and OER Studies in 0.1 M KOH

**Figure 5:**
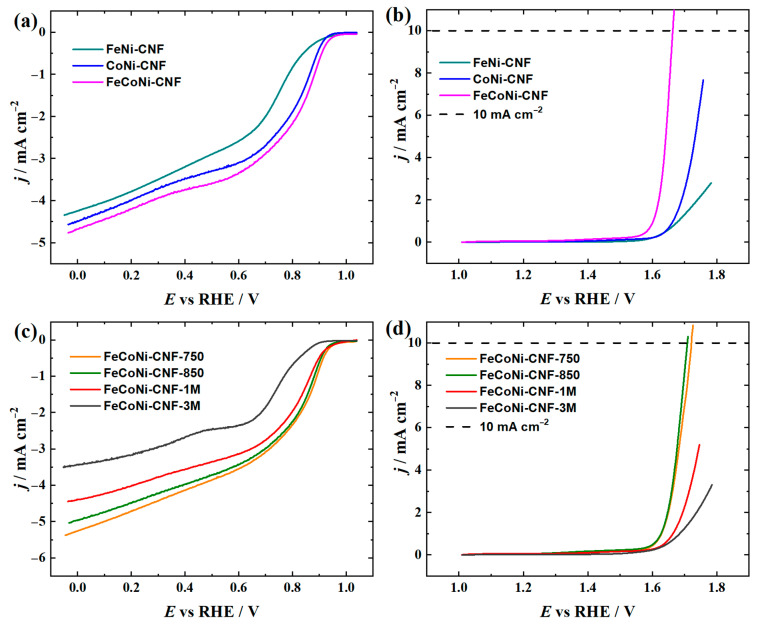
RDE polarisation curves for (**a**,**c**) O_2_ reduction and (**b**,**d**) O_2_ evolution on differently prepared CNF catalyst-coated glassy carbon electrodes in (**a**,**c**) O_2_-saturated or (**b**,**d**) Ar-saturated 0.1 M KOH (*ω* = 1900 rpm, *ν* = 10 mV s^–1^).

The bifunctional ORR and OER activity of the CNF catalyst was firstly evaluated in 0.1 M KOH solution using the RDE voltammetry (Figure 5 and Table 3). The electrocatalytic activity towards the ORR can be described using the half-wave potential (*E*_1/2_), which was most positive in the case of three TM Pc compound functionalized materials pyrolyzed at 800 and 850 °C (*E*_1/2_ = 0.77 V). The FeCoNi-CNF-materials coated electrodes exhibited higher oxygen reduction current density in the studied potential range compared to two TM Pc functionalized catalysts. The benefit of combining Fe-Nx and Co-Nx sites in the nanocarbon catalyst is generally recognised to be advantageous for achieving higher ORR activity compared to single TM-Nx-containing materials [15,17,75]. Moreover, in a study by Sonkar et al., the functionalization of reduced graphene oxide catalyst with NiPc provided enhanced oxygen reduction performance and a 4e^−^ ORR pathway [52]. Therefore, all three TM-Nx sites can be considered beneficial for O_2_ reduction, which could explain the superiority of tri-doped catalysts compared to the dual-doped ones within this study. In general, the FeCoNi-CNF exhibits similar ORR performance to CoPc and FePc dual-doped IL-containing PAN-based CNF materials in our previous investigation [44]. Moreover, the latter catalysts showed considerably higher ORR performance after the acid leaching procedure [44], which then achieved the *E*_1/2_ values similar to that of highly active CoPc and FePc dual-doped CNT catalyst [71]. Surprisingly, in the present investigation, the acid treatment had a deteriorating effect according to the oxygen reduction current density in case of both studied leaching solutions with 1 M and 3 M concentrations.

The OER activity investigations revealed that the tri-doped FeCoNi-CNF was the only material that achieved the 10 mA cm^−2^ compared to the dual-doped catalyst (Figure 5, Table 3). The lowest potential value for OER current density at 10 mA cm^−2^ (*E*_10_) was obtained in the case of FeCoNi-CNF material pyrolyzed at 800 °C. The use of 50 °C lower or higher carbonisation temperature resulted in ca. 50–70 mV higher *E*_10_ values. The acid leaching of FeCoNi-CNF catalyst caused a considerable decrease in the OER activity, as this procedure removes TM oxides, which are considered to be the main active species for OER in the present nanocarbon catalysts [37,68]. The oxides of all used TM (Ni, Fe, Co) compounds have been reported to exhibit high activity towards the OER [40,66,67,70,76,77], which could explain the best performance of tri-doped catalyst in this work due to the synergy of all three TM oxides. Moreover, as ca. 8 at% of surface O was detected for FeCoNi-CNF by XPS, the oxygen-containing functional groups on the CNF surface (e.g., C=O) can also enhance the OER performance as reported by Lu et al. in a study of oxidized CNT catalysts [78].

The ORR/OER reversibility (∆*E*), which is the difference between the *E*_1/2_ and *E*_10_ values, can be used for assessing the suitability of a material to be employed as a bifunctional oxygen catalyst. In the present work, the lowest ∆*E* value of 0.89 V was obtained in the case of FeCoNi-CNF catalyst, which is ca. 100 mV higher compared to the ∆*E* value found in the case of polymer-derived ceramic nanowire catalysts with metallic silicide tips in our previous investigation of RZAB air electrode catalysts [46]. The dual-doped TM Pc functionalized CNT-based materials have shown even better ∆*E* values (0.76–0.84 V) in recent papers [40,71]. The superior bifunctional activity in the case of CNT-based catalysts could be due to a higher specific surface area available for accommodation of catalytically active sites on the multi-walled CNTs with ca. 10 nm diameter (Nanocyl SA) compared to CNFs with a mean diameter of ca. 550 nm (Figure 1).

**Figure 6:**
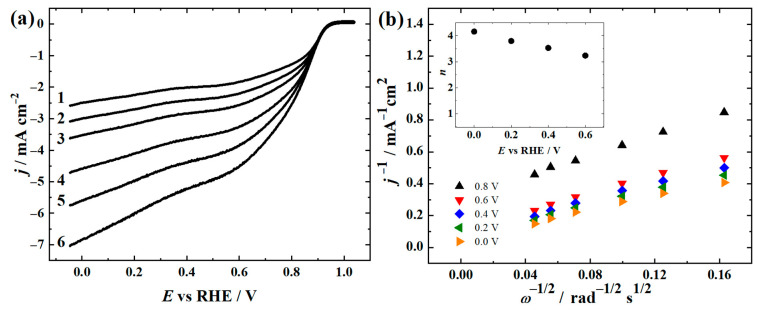
(**a**) RDE voltammetry curves for oxygen reduction on FeCoNi-CNF catalyst in O_2_-saturated 0.1 M KOH (*ν* = 10 mV s^–1^) at (1) 360, (2) 610, (3) 960, (4) 1900, (5) 3100, and (6) 4600 rpm. (**b**) Koutecky–Levich plots for O_2_ reduction in 0.1 M KOH derived from the RDE polarisation curves in (**a**). The inset shows the potential dependence on the value of *n*.

The ORR on FeCoNi-CNF catalyst was studied in more detail using different rotation rates in 0.1 M KOH solution (Figure 6). The Koutecky–Levich (K-L) plots derived from the RDE voltammetry curves show that the ORR proceeds via the mixed mass-transport and kinetic limitation conditions as the extrapolated K-L lines would not pass the origin of the axis in the studied potential range. The electron transfer number (*n*) was calculated by the K-L equation [79] using the parameters taken from our previous investigation [37]. The *n* value was between 3 and 4, indicating that the ORR proceeds mainly via the 4e^−^ route, which is favoured due to the higher energy efficiency compared to the 2e^−^ pathway [5].

**Figure 7:**
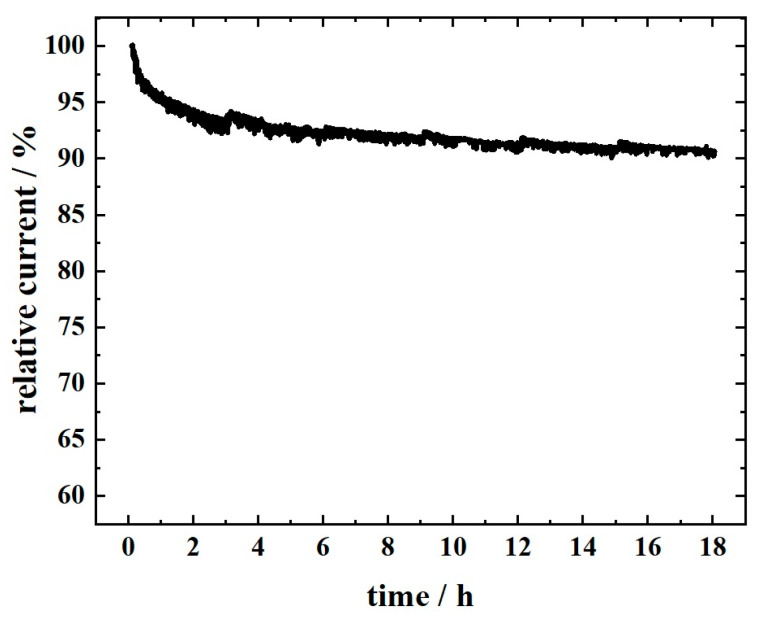
Chronoamperometry stability testing at 0 V vs. RHE for ORR on D-MN_4_-CNF-IL-A coated GC electrode in O_2_-saturated 0.1 M KOH at 960 rpm. Loading: 0.2 mg cm^−2^.

As the stability of the catalyst material is of utmost importance, chronoamperometry ORR stability testing was performed with FeCoNi-CNF catalyst, similar to the procedure in our previous investigation (Figure 7) [44]. The production of peroxide due to the observed partial 2e^−^ pathway (Figure 6b) seems not to have a remarkable effect on the catalyst material durability, as ca. 10% decrease in the relative current was observed during the 18 h hydrodynamic chronoamperometry test. Therefore, the FeCoNi-CNF material is found to be suitable for the application as a catalyst at the RZAB air electrode.

### 3.3. Zn–Air Battery Testing

**Figure 8:**
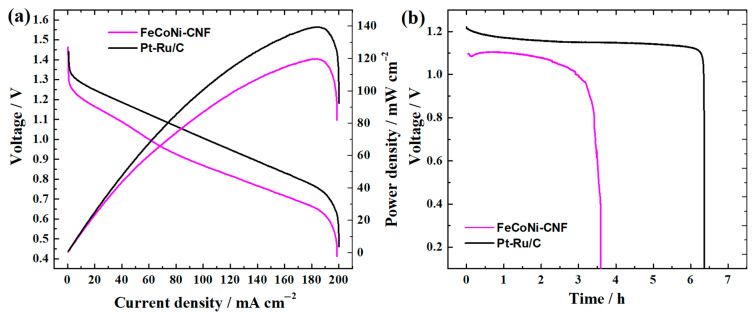
Primary Zn–air battery testing with FeCoNi-CNF and Pt-Ru/C catalyst-coated air cathodes: (**a**) discharge polarisation and power density curves recorded using a discharge current rate of 10 mA min^−1^; (**b**) complete discharge at constant 20 mA cm^−2^ as a function of time.

The ∆*E* value can show the suitability of the catalyst to be employed at the RZAB air electrode. Therefore, the FeCoNi-CNF material with lowest ∆*E* value was chosen for this application. After assembling the RZAB, the open circuit voltage (OCV) of 1.51 V was recorded, which is closer to the theoretical value of the Zn–air system (1.65 V [80]) as compared to the OCV of 1.45 V obtained in the case of commercial Pt-Ru/C. Firstly, the primary battery performance was evaluated by recording the battery discharge polarisation curve (Figure 8a). During the polarisation at 184 mA cm^−2^, the maximum power density (*P*_max_) of 120 mW cm^−2^ was obtained, which is 86% of the *P*_max_ measured for Pt-Ru/C air electrode catalyst (*P*_max_ = 139 mW cm^−2^). For comparison, similar *P*_max_ values between 115 and 125 mW cm^−2^ have been reported for different N-containing nanocarbon air electrode catalyst materials co-doped with two [81,82,83,84,85] or three TMs [27,30]. Nevertheless, it should be mentioned that the Zn-electrode area can play a crucial role in the measured *P*_max_ value, which highlights the necessity to include the comparison curve with commercial PGM catalyst [47,80]. Also, the total discharge curve at 20 mA cm^−2^ was recorded for primary batteries with Pt-Ru/C and FeCoNi-CNF air electrodes to calculate the specific capacity according to the amount of consumed Zn electrode (Figure 8b) [46]. Specific capacities of 695 and 407 mAh g_Zn_^−1^ were obtained for Pt-Ru/C and FeCoNi-CNF, respectively, while the theoretical specific capacity is 820 mAh g_Zn_^−1^ [4]. Based on these results, primary ZAB with FeCoNi-CNF air cathode can achieve 50% of the theoretical specific capacity.

**Figure 9:**
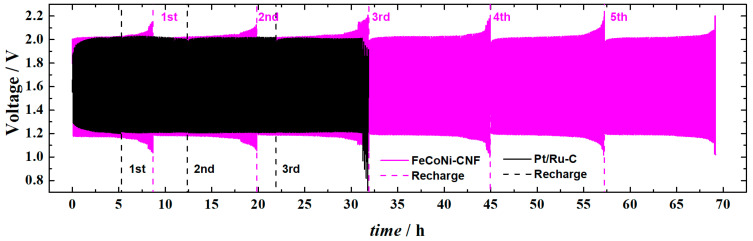
Rechargeable Zn–air battery testing with FeCoNi-CNF and Pt-Ru/C catalyst-coated air electrodes; consecutive charge and discharge cycles using 5 mA cm^−2^ for RZAB are shown together with the indication at which point in time the mechanical recharges were performed.

The secondary ZAB (i.e., RZAB) testing was performed to evaluate the FeCoNi-CNF as a bifunctional electrocatalyst for both ORR and OER. Pt-Ru/C was employed at the air electrode of RZAB in otherwise the same battery configuration (Figure 9). During continuous 5 mA cm^−2^ charge and discharge cycles (10 min per cycle), the RZAB eventually stops working due the formation of isolating ZnO layer at the Zn electrode. The battery can be mechanically recharged by replacing the Zn electrode and electrolyte, after which the RZAB operation can be continued [38,47]. Round-trip efficiencies of 58 and 60% were calculated for FeCoNi-CNF and Pt-Ru/C air electrode-based RZAB, respectively, at the 15th and also at the 25th hour of operation, showing the good stability of both bifunctional electrocatalysts up to 31 h. For comparison, the round-trip efficiency with FeCoNi-CNF air electrode is higher compared to the NiCo_2_O_4_ blended with N-doped graphene nanoribbon-based catalysts exhibiting 55% during 5 mA cm^−2^ cycling in an earlier investigation [66]. Herein, the Pt-Ru/C lost the catalyst activity after 31 h of cycling operation, as shown by the rapid decrease in battery discharge voltage. Mechanical recharge did not restore the battery performance. In a similar manner, abrupt voltage loss during discharge cycles has been previously reported for Pt/C + RuO_2_ composite air electrode catalyst-based RZABs [86]. No such decrease in activity was witnessed in the case of FeCoNi-CNF during the whole 68 h operation time, and the same round-trip efficiency of 58% was also registered at the 65th hour of operation. Additionally, the FeCoNi-CNF air electrode RZAB needed only two mechanical recharges in the same timeframe that Pt-Ru/C needed three.

These observations show that FeCoNi-CNF is the more suitable RZAB air-electrode than Pt-Ru/C in terms of cycling durability and also material cost comparison. More specifically, the rough estimation for the final FeCoNi-CNF preparation cost in comparison with the commercial Pt-Ru/C is provided in the Supplementary Material (Table S1). One can see that the total cost of FeCoNi-CNF (considering precursor components, their losses during the catalyst preparation, and equipment and production costs) is 23% compared to the cost of Pt-Ru/C. It should be noted that this value is obtained for two times higher catalyst loading of NPMC compared to Pt-Ru/C at the ZAB air electrode. The costs for equipment, labour, and electricity are calculated for a laboratory scale and could be further reduced in the case of industrial production volumes and co-production possibilities.

Furthermore, this is the first work describing the application of triple TM Pc-co-doped electrospun CNF-based bifunctional catalyst at the RZAB air electrode. The comparison with other recently reported TM-based NPMC air electrodes is shown in Table 4. One can observe that the bifunctional activity for both oxygen reactions and the RZAB performance properties for FeCoNi-CNF are rather similar to the ones of two different triple TM co-doped NPMC air electrodes prepared in the investigations by Wang et al. (Zn_0.4_Ni_0.6_Co_2_O_4_/NCNTs) [26] and Bejar et al. (Mn_0.5_Ni_0.5_Co_2_O_4_ 3DOM) [30]. Despite not meeting the highest ZAB performance reported for several TM-based NPMC air electrode catalysts listed in Table 4, the results obtained herein show that the first report for triple TM Pc-co-doped electrospun CNF-based catalyst (FeCoNi-CNF) is still an important step in the development of widely applicable NPMC for the RZAB air electrode catalyst.

## 4. Conclusions

In the present investigation, the preparation and characterization of Fe, Co, and Ni phthalocyanine tri-doped electrospun carbon nanofibre-based electrocatalyst material (FeCoNi-CNF) was reported. The three different TM Pc compound doped catalyst was found to be more active towards the ORR and OER compared to the corresponding CNF materials that were prepared using only two TM Pc combinations, Ni/Fe and Ni/Co. The pyrolysis temperature was optimised, and acid leaching was found to be unbeneficial for enhancing bifunctional ORR/OER activity. Physical characterization of FeCoNi-CNF revealed the highly defective graphitic CNF structure with the fibre diameter around 350–750 nm and uniform distribution of all TMs. The half-cell investigations in 0.1 M KOH solution showed the ∆*E* value of 0.89 V for FeCoNi-CNF material. The application of FeCoNi-CNF at the air electrode of primary ZAB showed the *P*_max_ of 120 mW cm^−2^ and specific capacity of 407 mAh g_Zn_^−1^. In RZAB configuration, the FeCoNi-CNF air electrode was found to be superior compared to the commercial Pt-Ru/C catalyst in terms of operational durability between recharges and considerably higher total life-time of at least 68 h.

## Figures and Tables

**Table 1:** Designation and content of precursor materials (wt%) used for the electrospinning of different fibre materials for the catalyst preparation.

Catalyst Designation	Electrospinning Solution Components
FeNi-CNF	10% [BMIm]Ac; 58% PAN; 16% FePc; 16% NiPc
CoNi-CNF	10% [BMIm]Ac; 58% PAN; 16% CoPc; 16% NiPc
FeCoNi-CNF	10% [BMIm]Ac; 58% PAN; 10.6% FePc; 10.6% CoPc; 10.6% NiPc

**Table 2:** Elemental composition (at%) of FeNi-CNF, CoNi-CNF, and FeCoNi-CNF catalyst materials determined by XPS (Figure 2). Relative content (%) of various N species as determined by XPS for FeNi-CNF, CoNi-CNF, and FeCoNi-CNF catalyst (insets in Figure 2).

Catalyst Material	C	N	O	Fe	Co	Ni
FeNi-CNF	83.1	9.4	6.7	0.3	0	0.5
CoNi-CNF	82.4	7.7	9.2	0	0.4	0.3
FeCoNi-CNF	83.6	7.5	8.1	0.1	0.4	0.3
**Catalyst material**	**imine**	**pyridinic-N**	**TM-Nx**	**pyrrolic-N**	**graphitic-N**	**bulk N-H**
FeNi-CNF	1	47	6	32	11	3
CoNi-CNF	2	46	11	29	10	2
FeCoNi-CNF	3	45	11	29	9	3

**Table 3:** The ORR half-wave potential (*E*_1/2_), potential at the OER current density of 10 mA cm^−2^ (*E*_10_), and ORR/OER reversibility (∆*E* = *E*_1/2_ − *E*_10_) values for different catalyst-coated GC electrodes obtained from Figure 5.

Electrode	*E*_1/2_ (V)	*E*_10_ (V)	∆*E* (V)
FeNi-CNF	0.68	-	-
CoNi-CNF	0.76	-	-
FeCoNi-CNF	0.77	1.66	0.89
FeCoNi-CNF-750	0.76	1.72	0.96
FeCoNi-CNF-850	0.77	1.71	0.94
FeCoNi-CNF-1M	0.78	-	-
FeCoNi-CNF-3M	0.71	-	-

**Table 4:** Comparison of ORR and OER half-cell performance results in 0.1 M KOH and RZAB characteristics for different TM-based NPMC air electrodes.

Catalyst	*E*_1/2_, V	*E*_10_, V	Δ*E*, V	OCV, V	*P*_max_, mW cm^−2^	Specific Capacity, mAh g_Zn_^−1^	Stability, Time @j (mA cm^−2^)	Ref.
FeCoNi-CNF	0.77	1.66	0.89	1.51	120	407	68 h @5	This work
Zn_0.4_Ni_0.6_Co_2_O_4_/NCNTs	0.78	1.64	0.86	1.48	109	690	100 cycles @25	[26]
Mn_0.5_Ni_0.5_Co_2_O_4_ 3DOM	0.76	1.63	0.87	1.38	117	-	21 h @10	[30]
5 wt%-Ni/PDC	0.62	1.61	0.99	1.24	59	608	200 h @5	[46]
FeNi/N-CPCF-950	0.87	1.59	0.72	1.48	161	751	640 h @10	[23]
FeNiN-MWCNT	0.87	1.59	0.72	1.45	84.5	-	48 h @1	[37]
Co_3_O_4_/Fe_2_O_3_NAs@CNFs	0.80	1.60	0.80	1.53	256	-	40 h @10	[70]
Ni|MnO/CNF	0.83	1.59	0.76	1.56	139	-	120 h @10	[24]
CoFe-LDH@FeCo NPs-N-CNTs	0.89	1.57	0.68	1.51	116	799	100 h @10	[82]
H-Co@FeCo/N/C	0.91	1.61	0.70	1.45	125.2	-	200 h @10	[81]
FeCoNi-NC	0.89	1.54	0.65	1.52	315	804	100 h @50	[28]
Fe/Co/Zn-CN_ZIF_	0.85	1.58	0.73	1.46	157	769	137 h @5	[32]
V_o_-CoFe/CoFe_2_O_4_@NC	0.86	1.59	0.73	1.53	140	775	45 h @10	[87]
NiCo_2_O_4_(90):GNRN(10)	0.76	1.62	0.86	1.43	69	702	96 h @2	[66]

## Data Availability

The data presented in this study are available on request from the corresponding author.

## Supplementary Materials

The following supporting information can be downloaded at: https://www.mdpi.com/article/10.3390/ma16134626/s1, Figure S1: (a,b) SEM micrographs of FeCoNi-CNF-1M catalyst with different magnifications; Table S1: Cost estimation for 10 g of FeCoNi-CNF catalyst considering the price of the precursor materials, approximate material loss during mixing and electrospinning procedures (5%), the pyrolysis yield (52%), and equipment and production costs. The cost of 5 g of Pt-Ru/C (50:25:25, Alfa Aesar) catalyst, which was employed for primary and secondary ZAB performance comparison, is also listed. The prices for the products are given with internet geolocation in Estonia as of 11 June 2023 without VAT.
