# Supplementary material for: Iron, Cobalt, and Nickel Phthalocyanine Tri-Doped Electrospun Carbon Nanofibre-Based Catalyst for Rechargeable Zinc–Air Battery Air Electrode
Source: Materials (Basel). 2023 Jun 27;16(13):4626. doi: 10.3390/ma16134626 (PMC10342803; doi:10.3390/ma16134626)
Supplement: Supplementary file 1 [file materials-16-04626-s001.zip › materials-2437750-supplementary.pdf]

# Supplementary Material

# Iron, Cobalt, and Nickel Phthalocyanine Tri-Doped Electrospun Carbon Nanofibre-Based Catalyst for Rechargeable Zinc-Air Battery Air Electrode

**Kaur Muuli [1], Rohit Kumar [1], Marek Mooste [1,*], Viktoria Gudkova [2], Alexey Treshchalov [3], Helle-Mai Piirsoo [3], Arvo Kikas [3], Jaan Aruväli [4], Vambola Kisand [3], Aile Tamm [3], Andres Krumme [2], Prabu Moni [5], Michaela Wilhelm [5] and Kaido Tammeveski [1]**

1 Institute of Chemistry, University of Tartu, Ravila 14a, 50411 Tartu, Estonia
2 Department of Materials and Environmental Technology, Tallinn University of Technology, Ehitajate tee 5, 19086 Tallinn, Estonia
3 Institute of Physics, University of Tartu, W. Ostwald Str. 1, 50411 Tartu, Estonia
4 Institute of Ecology and Earth Sciences, University of Tartu, Vanemuise 46, 51014 Tartu, Estonia
5 University of Bremen, Advanced Ceramics, Am Biologischen Garten 2, IW3, 28359 Bremen, Germany
* Correspondence: marek.mooste@ut.ee

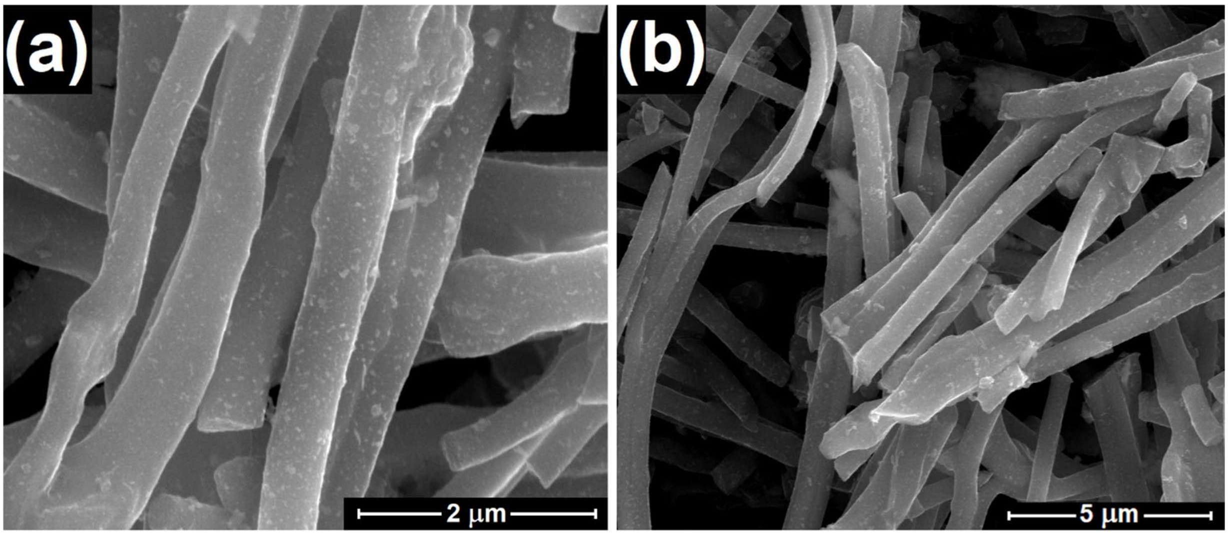


**Figure S1.** (a, b) SEM micrographs of FeCoNi-CNF-1M catalyst with different magnifications.

**Table S1.** Cost estimation for 10 g of FeCoNi-CNF catalyst considering the price of the precursor materials, approximate material loss during mixing and electrospinning procedures (5%), the pyrolysis yield (52%), equipment and production costs. The price for 5 g of Pt-Ru/C (50:25:25, Alfa Aesar) catalyst, which was employed for primary and secondary ZAB performance comparison is also listed. The prices for the products are given with internet geolocation in Estonia as of 11 June 2023 without VAT.

| Precursor material | CAS number | Product number | Pack size | Price | Total weight | Required amount | Cost per amount |
|---|---|---|---|---|---|---|---|
| DMF[a] | 68-12-2 | 319937-2.5L | 2.5L | €239.00 | 2370 g | 52.20 g | €5.30 |
| PAN[a] | 25014-41-9 | 181315-50G | 50 g | €155.00 | 50 g | 5.80 g | €18.00 |
| IL[a] | 284049-75-8 | 39952-1KG-F | 1 kg | €1290.00 | 1000 g | 1.00 g | €1.30 |
| FePc[a] | 132-16-1 | 379549-10G | 10 g | €149.00 | 10 g | 1.06 g | €15.80 |
| CoPc[a] | 3317-67-7 | 307696-50G | 50 g | €719.00 | 50 g | 1.06 g | €15.25 |
| NiPc[a] | 14055-02-8 | 360635-5G | 5 g | €149.00 | 5 g | 1.06 g | €31.60 |

| Catalyst material | Influence factor on the material cost | Final cost |
|---|---|---|
| FeCoNi-CNF | Cost of all precursor materials for 10 g of electrospun fibre material | €87.25 |
| FeCoNi-CNF | Cost of labor, consumables, equipment, and energy for 10 g of fibre material | €37.00 |
| FeCoNi-CNF | Combined cost of precursors and production (€124.25) including material loss (5%) | €130.80 |
| FeCoNi-CNF | Consideration of the pyrolysis yield (52%) for FeCoNi-CNF (10 g) | €251.52 |
| Pt (50%), Ru (25%) on high surface area advanced carbon support (Stock No. 47371.06, 5 g)[b] | | €1092.00 |

[a]Product information obtained from the webpage https://www.sigmaaldrich.com/.
[b]Product information obtained from the webpage https://www.alfa.com/.
